# Supplementary material for: MetaRibo-Seq measures translation in microbiomes
Source: Nat Commun. 2020 Jun 29;11:3268. doi: 10.1038/s41467-020-17081-z (PMC7324362; doi:10.1038/s41467-020-17081-z)
Supplement: Supplementary file 10 — Supplementary Data 7 [file 41467_2020_17081_MOESM10_ESM.zip › File2/Confidence_VeryHigh_Taxonomy/312841_out.krona.html]

Javascript must be enabled to view this page.

members
magnitude
magnitudeUnassigned
count
unassigned
taxon
rank

312841\_out

4

superkingdom
2
4

4
976
phylum

4
200643
class

order
171549
4

2
family
171552

370804
species
1

SRS049446\_contig\_number\_6258

1
genus
838

159272
species

SRS149325\_contig\_number\_1840
1

815
family
1

1
genus
816


SRS140492\_contig\_number\_26467
1
818
species

2005525
family
1

1
genus
375288

species
46503
1

SRS098073\_contig\_number\_12108
